# Clinical and histological evaluation of alloderm GBR and BioOss in the treatment of Siebert's class I ridge deficiency

**DOI:** 10.4103/0972-124X.44099

**Published:** 2008

**Authors:** Sabitha Sudarsan, K. V. Arun, M. S. Priya, Ramya Arun

**Affiliations:** *Department of Periodontics, Ragas Dental College and Hospital, Uthandi, India*; 1 *Department of Periodontics, Tagore Dental College and Hospital, Chennai, India*

**Keywords:** Alloderm, Bio-Oss, guided bone regeneration

## Abstract

Complete prosthetic rehabilitation using implants require the presence of adequate dimensions of alveolar bone. Ridge augmentation procedures include the use of guided bone regeneration (GBR) procedures where the barrier membrane provides cell occlusion and space for the regenerating tissues. Alloderm GBR has been introduced for the purpose of augmenting bone and has been postulated to have the additionally ability to integrate into soft tissues. Twenty-two patients with Siebert's class I ridge deficiency were treated with BioOss and Alloderm GBR and followed up for a period of nine months. Significant increase in ridge dimensions of both hard and soft tissues were observed at six months period itself, suggesting that it as an effective method of augmenting deficient ridges.

## INTRODUCTION

Implant related prosthesis has become an integral part of the treatment strategy for replacement of missing teeth. Even with the advancements in the technology involving the manufacture of implants and surgical methodology, the residual alveolar ridge plays a major role in the long term success of this prosthesis.[1] The availability of an ideal volume of bone has been shown to significantly increase the esthetic and functional acceptability of implants.[2]

Alveolar ridge deformities are commonly associated with traumatic extraction of teeth, periodontal disease, developmental defects or as a result of resorption occurring due to lack of teeth. Deficient ridges are treated either through augmentation of hard or soft tissues or combination of both. An increasing number of surgical techniques to reconstruct localized ridge deformities have been presented in the literature since Abrams first offered a method to reconstruct ridge defects in 1971[3] The necessity for augmenting the volume of bone is obvious in that implant stability requires optimum contact of the implant with bone over a sufficiently large surface area to ensure good osseointegration.[4] The soft tissue characteristics are also equally important in that adequate keratinised mucosa is known to absorb mechanical stresses and retard the inflammatory spread.

Bone replacement grafts have been used to augment deficient ridges for the past two decades through the mechanism of osteogenesis, osteoinduction and osteoconductuon. Autogenous grafts are still considered the gold standard because of their ability to act through all three mechanisms, but the volume of bone available and postoperative morbidity are potential problems. Anorganic bone matrix (BioOss) is devoid of the protein content of bone while retaining its three dimensional mineralized network. It has been used in periodontal defects; ridge augmentation and sinus lift procedures. Regardless of the type of bone replacement graft used, the material does not prevent the soft tissue from entering the wound space, thereby affecting the regenerative process.[5]

Use of barrier membrane prevents the gingival soft tissue from contaminating the wound space, thereby enhancing the regenerative potential. AlloDerm was originally developed as a skin allograft for burn patients.[6] It was prepared from autogenous sources following cell lysis and the collagen matrix has been used in the treatment of gingival recession[7] and GBR procedures.[8]

The aim of this study is to evaluate the effect of anorganic bone material and alloderm GBR in the treatment of Siebert's class I ridge defect both clinically and histologically.

## MATERIALS AND METHODS

### Study design

Twenty-two patients in the age group 29-47 years (14 males and 8 females) selected from the Outpatient Department of Periodontics, Ragas Dental College and Hospital, Chennai, participated in this clinical trial. The patients reported with edentulous sites with Seibert's class I ridge defect, caused by at least one missing tooth. Patients with previous history of systemic diseases, pregnant and lactating women and smokers were excluded from the study. Informed consent was obtained from all the patients included in the study.

Patients were treated with Bio-Oss™ and AlloDerm GBR. They were reassessed post-operatively and reviewed at 2 weeks, 6 and 9 months. Clinical examinations were performed at follow-up visits to check for complications including infection, inflammation, wound dehiscence and resorption.

### Clinical parameters

**Figure 1:**
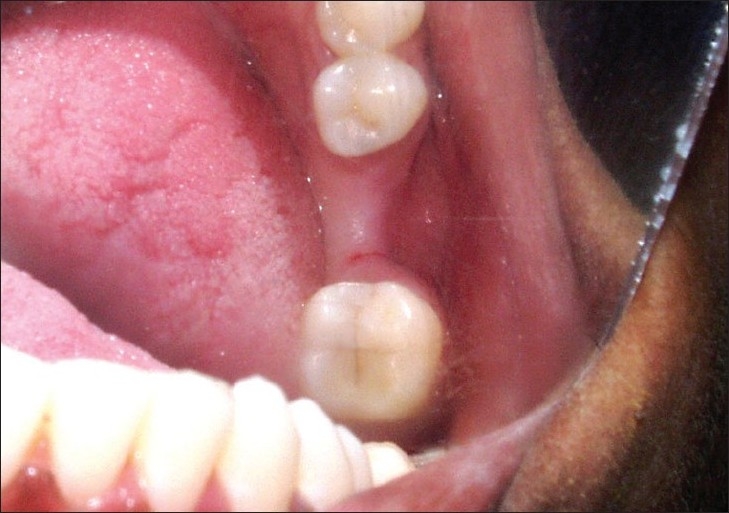
Siebert's class I ridge defect in 36 region (Preoperative)

Each case was documented with photographs and study models at baseline [Figure 1], six and nine months [Figure 6]. Clinical recordings included the measurement of the total ridge width, soft tissue thickness and bone width at baseline, six and nine months post operatively at fixed reference points: mid-point of the ridge, a point 2mm from the mid-point and a point 4 mm from the mid-point. Horizontal component of the ridge defect (bucco-lingual dimension) was measured using a boley gauge. Soft tissue thickness was measured on the buccal and the lingual aspect using endodontic reamers. The bone width was calculated by subtracting the total soft tissue thickness from the total ridge width. These measurements were made separately at both the 2 mm and 4 mm level from the crest of the alveolar bone.

**Figure 2:**
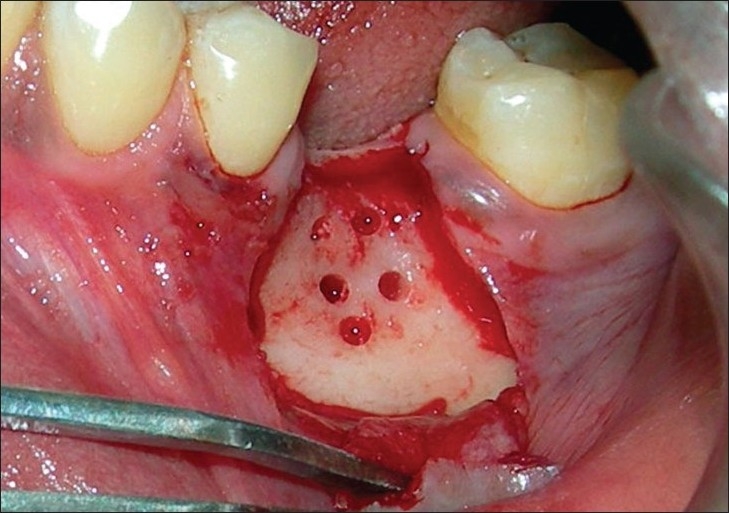
Full thickness flap raised and decortication holes placed (Note the careful preservation of interdental papilla)

**Figure 3:**
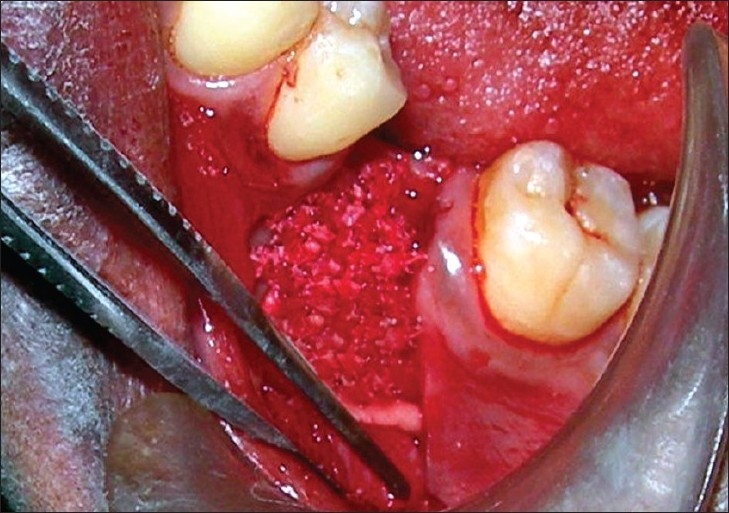
Anorganic bone graft (BioOss) placed to cover the defect

**Figure 4:**
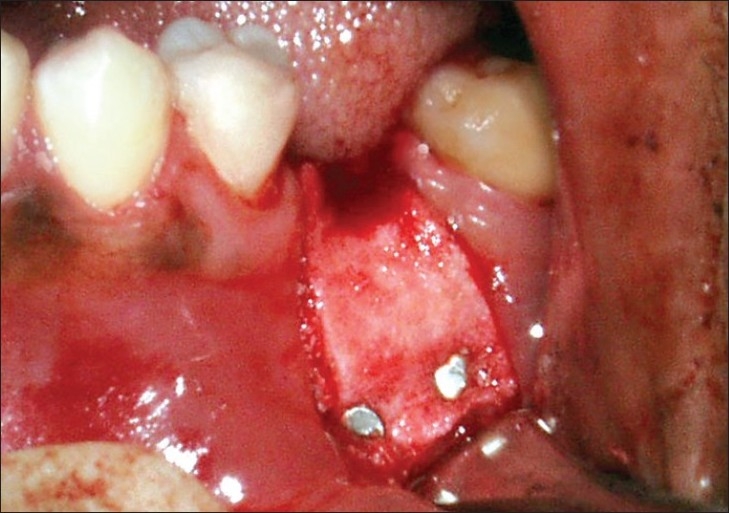
Alloderm GBR membrane placed over the bone graft and stabilised with autotacks

**Figure 5:**
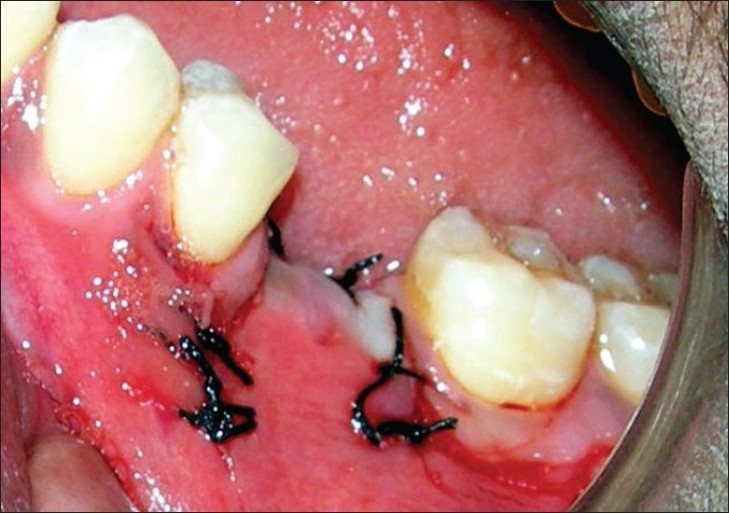
Flap approximated with silk sutures

**Figure 6:**
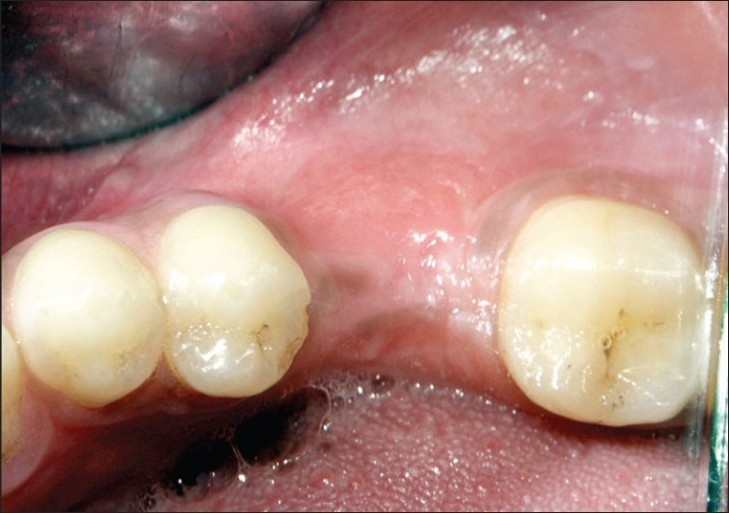
Nine months postoperative

### Surgical procedure

Patients were anesthetized with 2%lidocaine with epinephrine 1:80,000. Horizontal incisions were made slightly lingual to the midcrestal region and vertical incisions were made on the buccal surface from the mesial and distal extents of the horizontal incision extending to the mucogingival junction. The vertical incisions were designed to diverge as much as possible. Care was taken to preserve the interdental papilla. A full thickness mucoperiosteal flap was reflected on the buccal side and a pouch was created on the lingual side to facilitate insertion of the barrier membrane.

Approximate length and width of the membrane required was obtained using a sterile tin foil template cut to the size of the defect. Intramarrow cortical perforations were made at the recipient site at slow speed with copious saline irrigation [Figure 2]. This was done to open the marrow cavity as it is a source of angiogenic and osteogenic cells. It activates bone formation by the release of local and other bone-inducing factors. Bio-Oss™ (Geistlich Pharma AG) cancellous granules (0.25 - 1.0mm microns) were then mixed with patient's blood and placed on the prepared recipient bed covering the decortication site completely [Figure 3].

AlloDerm™ GBR membrane (Biohorizons, Inc.) was trimmed to the appropriate size of the template and then rehydrated with sterile saline making it soft and pliable throughout. AlloDerm GBR membrane had two sides- one corresponding to the basement membrane and the other, the connective tissue side. The membrane was placed in the surgical site with its connective tissue side in direct contact with the undersurface of the flap. Bone graft was covered with the rehydrated AlloDerm GBR membrane that extended at least 3 mm beyond the graft border in all directions. Membrane was secured in position using stabilisation screws (auto tacks) to prevent micromobility [Figure 4]. The flap was coronally repositioned for complete wound coverage without tension. Primary closure was then obtained using a 3.0 black silk suture [Figure 5]. Periodontal dressing was done with Coe-Pack.

Patients were advised appropriate medications and chlorhexidine mouth rinse b.i.d. for 3 weeks. All patients were reviewed at 1 week postoperatively and oral hygiene reinforcement was done. Sutures were removed at 3 week postoperative appointment.

### Histological evaluation of bone

Bone specimen was collected from the site of GBR procedure during the time of implant placement. Bone was collected from the osteotomy site using trephine burs and processed histologically.

The tissue was fixed in 10% formalin and then decalcified in 5% nitric acid. It was washed with distilled water and then immersed in 10% formalin for a period of 24 hours.

### Processing of tissue

The tissue was subjected to two washes in xylene each one hour followed by immersion in 90% isopropyl alcohol for one hour and 70% isopropyl alcohol for another one hour. Then it was impregnated in paraffin wax and embedded in fresh paraffin wax. 4µ thick sections of the tissue was made with microtome and mounted on slides coated with egg albumin.

### HandE staining

The sections were washed in 100% and then 70% isopropyl alcohol for 5 minutes each. Then two washes with xylene each 5 mins. It was then rinsed with distilled water for 5 minutes, immersed in hematoxylin for 5 minutes, rinsed again with distilled water. The sections were then immersed in 1% acid alcohol, rinsed with distilled water and finally immersed in eosin one dip. The sections were then mounted in DPX and viewed under light microscope (10× and 40×).

### Statistical analysis

Statistical analysis was performed using the one-way ANOVA test to evaluate the overall significance at different time intervals for ridge dimensions at 2 mm and 4mm from the alveolar crest. Also, Student Newman Keuls test was used to evaluate the significance within the groups at different time intervals.

P< 0.001 was considered as significant at 1% level of significance. (One-way ANOVA). In the present study *P* <0.05 was considered as significant at 5% level of significance. (Student Newman Keuls test)

Percentage in gain of ridge width, bone and soft tissue thickness was calculated by the following formula:

$$\%\mathrm{gain}\;=\;\frac{\mathrm{Post operative measurement}\;-\;\mathrm{Preoperative measurement}\;\times \;100}{\mathrm{Post operative measurement}}$$

## RESULTS

### Total ridge width at 2 mm and 4 mm level from the alveolar crest

**Figure 7:**
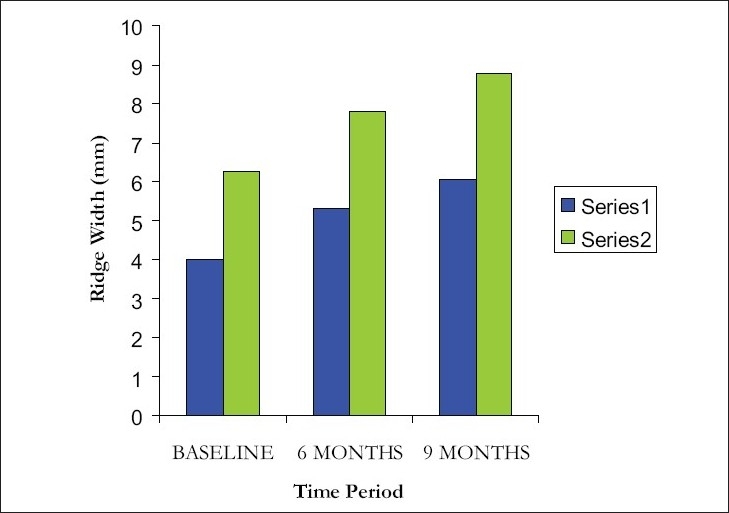
Comparison of ridge width at 2 mm and 4 mm level from alveolar crest at different intervals

At 2mm level, mean ridge width was measured at baseline as 4.00 ± 0.47 mm that increased to 5.30 ± 0.48 mm at the end of 6 months and 6.05 ± 0.69 mm at the end of 9 months. There was a percentage increase of 33% and 51% at 6 months and 9 months respectively. At 4mm level, mean ridge width was found to be 6.25 ± 0.42 mm at baseline, that increased to 7.80 ± 0.35 mm at the end of 6 months and 8.75 ± 0.42 mm at the end of 9 months [Figure 7]. There was a percentage increase of 25% and 40% at 6 months and 9 months respectively.

Overall comparison within groups and between groups was statistically significant at 1 % level. (*P* <0.001) The values were found to be significant when compared to the baseline and 6 months, baseline and 9 months, 6 and 9 months at 5% level of significance (*P* < 0.05).

### Soft tissue thickness at 2 mm and 4 mm from alveolar crest

**Figure 8:**
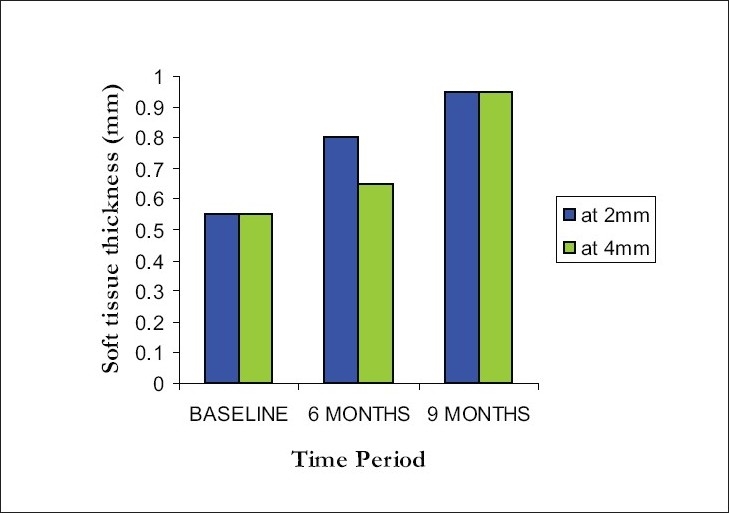
Comparison of soft tissue thickness at 2 mm and 4 mm level from alveolar crest at different intervals

At 2mm level, mean width of soft tissue thickness increased from 0.55 ± 0.16mm at baseline to 0.80 ± 0.26mm at 6 months and 0.95 ± 0.28mm at the end of 9 months. There was a percentage increase of 45% and 73% at 6 months and 9 months respectively. At 4mm level, mean width of soft tissue thickness increased from 0.55 ± 0.16mm at baseline to 0.65 ± 0.24mm at 6 months and 0.95 ± 0.28mm at the end of 9 months [Figure 8]. There was a percentage increase of 18% and 73% at 6 months and 9 months respectively.

Overall comparison within groups and between groups was statistically significant at 5 % level. (*P* < 0.0033 for 2 mm level and *P* < 0.0020 at 4 mm level) The values were found to be significant when compared to the baseline and 6 months, baseline and 9 months, at 5% level of significance (*P* < 0.05). But there was no statistical significance between 6 and 9 months period.

### Actual bone width at 2 mm and 4 mm level from the alveolar crest

**Figure 9:**
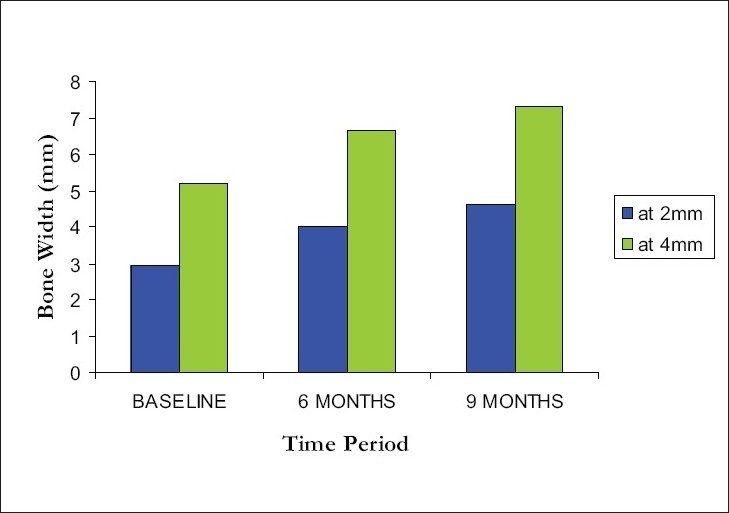
Comparison of bone width at 2 mm and 4 mm level from alveolar crest at different intervals

Similar to total ridge width, at 2mm level, mean bone width showed a marked improvement from 2.95 ± 0.50mm at baseline to 4.00 ± 0.53mm at 6 months and 4.60 ± 1.57mm at the end of 9 months. There was a percentage increase of 33% and 51% at 6 months and 9 months respectively. At 4mm level, the mean bone width was measured to be 5.20 ± 0.48 mm at baseline, that was augmented to 6.65 ± 0.41mm at 6 months and 7.30 ± 0.54 mm at the end of 9 months [Figure 9]. There was a percentage increase of 28% and 48% at 6 months and 9 months respectively.

Overall comparison within groups and between groups was statistically significant at 1 % level. (*P* < 0.001) The values were found to be significant when compared to the baseline and 6 months, baseline and 9 months, 6 and 9 months at 5% level of significance (*P* < 0.05).

### Hand E staining of bone tissue

**Figure 10:**
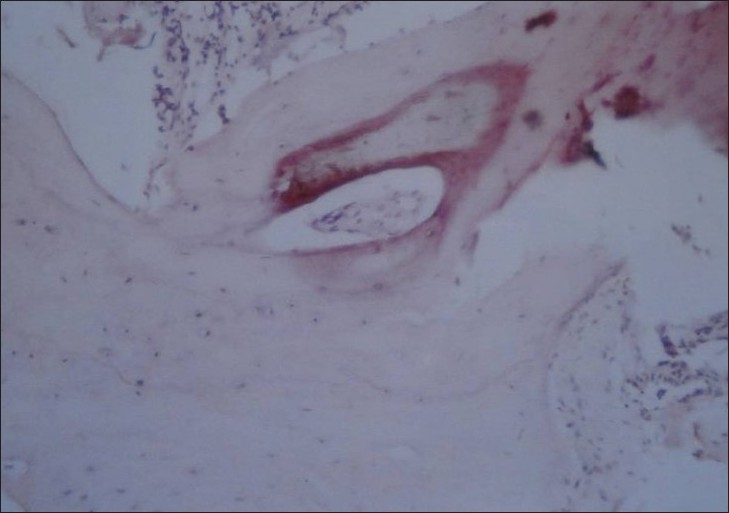
H & E staining of the regenerated bone tissue (10× view)

**Figure 11:**
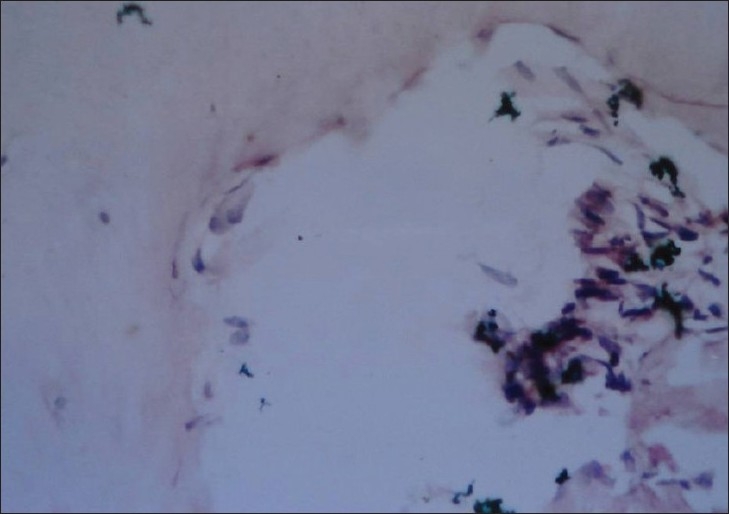
H & E staining of the regenerated bone tissue (40× view)

10× view shows spicules of vital lamellar bone with resting and reversal lines and numerous osteocytes within the lacunae [Figure 10]. The lamellar bone is associated with marrow connective tissue exhibiting inflammatory cells. 40× view shows osteoblasts rimming around the vital bone with few inflammatory cells [Figure 11.]

## DISCUSSION

The necessity to augment deficient alveolar ridges prior to implant placement is well recognised. Guided bone regeneration is an accepted method of augmenting bone today.[9] Lazzara is credited with the first reported use of GBR techniques with implants in immediate extraction sites.[10]

Ridge deficiencies have been classified according to their severity and dimension in which they are deficient.[11] The difficulty in increasing the vertical dimension of the ridge is well documented. As the predictability of regeneration has been shown to be much higher when there is only a bucco-lingual loss of tissue, class I ridge deficiency were chosen for this study.

Anorganic bone material used in this study was prepared from bovine sources. Its lack of antigenecity and good handling characteristics has been previously documented. Its three dimensional porous structure has been postulated to enhance cellular in-growth into the grafted area. Several studies are documented to bone formation after placement of anorganic bone material in periodontal defects, deficient ridges[12] and in sinus lift procedures.[13]

Alloderm GBR is a collagen based barrier membrane prepared from dermis that is processed to remove its cellularity and subsequently cryoprecipitated to remove its antigenecity. Structurally, it is similar to alloderm used for recession coverage and differs only in its thickness. Alloderm has a connective tissue and basement membrane side, the orientation of which is unimportant. However, in this study, as a rationale, the connective tissue side was placed facing the flap surface to facilitates its incorporation with the collagen fibers on the under surface of the flap.[14]

All the defects that were treated in this study showed an unremarkable healing process. Membrane exposure was minimised probably by the placement of tacks[15] and by obtaining sufficiently relaxed flap and secure suturing.[16]

There was a significant increase in the ridge dimension following the procedure at the six and nine month intervals. The greatest increase in ridge dimension was observed between baseline and six months when compared to nine month interval. Most studies documenting ridge augmentation have shown significantly greater formation of bone after six months of placing the graft. The results of this study are therefore in conformation with previous studies.[17]

Ridge measurement was taken at 2mm and at 4mm level separately so as to assess the overall improvement in the ridge dimension. The increase in ridge dimension level was significantly greater at 2mm level than at 4mm level. The measurements taken during baseline at 2mm level shows greater deficiency of bone when compared to 4mm level. Greater regeneration observed in areas of well defined osseous defects is in conformity with previous studies that have documented the same.[18]

The results of this study show that following treatment with BioOss and Alloderm GBR there was a significant increase in hard and soft tissue dimensions. The ability of alloderm to integrate with the gingival soft tissue is probably responsible for the significant improvement in the soft tissue dimensions. The increase in soft tissue dimension has not been previously reported with collagen barrier membranes. The improved soft tissue characteristic as observed in this study denotes that alloderm is a good candidate for use in GBR procedures. Long term results following use of alloderm in recession coverage procedures suggest that two year results were not fully satisfactory. Hence, long term studies of alloderm in guided bone regeneration need to be conducted to determine if the soft tissue characteristics remain stable over a long period of time.

The histological picture shows the presence of viable new bone formation with osteoblasts and a few inflammatory cells in the site indicating that there is actual new bone formation that is undergoing turnover and therefore ideal for implant placement.

## CONCLUSION

Alloderm GBR may be used as a barrier membrane for the treatment of class I ridge deficiency and may provide the additional advantage of improved soft tissue characteristics as well.
